# A global, empirical, harmonised dataset of soil organic carbon changes under perennial crops

**DOI:** 10.1038/s41597-019-0062-1

**Published:** 2019-05-13

**Authors:** Alicia Ledo, Jonathan Hillier, Pete Smith, Eduardo Aguilera, Sergey Blagodatskiy, Francis Q. Brearley, Ashim Datta, Eugenio Diaz-Pines, Axel Don, Marta Dondini, Jennifer Dunn, Diana Marisa Feliciano, Mark A. Liebig, Rong Lang, Mireia Llorente, Yuri Lopes Zinn, Niall McNamara, Stephen Ogle, Zhangcai Qin, Pere Rovira, Rebecca Rowe, José Luis Vicente-Vicente, Jeanette Whitaker, Qian Yue, Ayalsew Zerihun

**Affiliations:** 1 University of Aberdeen, Institute of Biological and Environmental Sciences, 23 St Machar Drive, Aberdeen, AB24 3UU UK; 2 Global Academy of Agriculture and Food Security, The Royal (Dick) School of Veterinary Studies and The Roslin Institute, Easter Bush Campus, Midlothian, EH25 9RG UK; 3 CEIGRAM-ETSIAAB, Technical University of Madrid, Madrid, 28040 Spain; 4 Institute of Agricultural Sciences in the Tropics (Hans-Ruthenberg-Institute), University of Hohenheim, Garbenstrasse 13, 70599 Stuttgart, Germany; 5 School of Science and the Environment, Manchester Metropolitan University, Chester Street, Manchester, M1 5GD UK; 6 Division of Soil and Crop Management, ICAR-Central Soil Salinity Research Institute, Karnal, Haryana 132001 India; 7 University of Natural Resources and Life Sciences, Vienna (BOKU), Institute of Soil Research, Peter-Jordan-Strasse 82, 1190 Vienna, Austria; 8 Thünen Institute of Climate-Smart Agriculture, Bundesallee 65, 38116 Braunschweig, Germany; 9 Energy Systems Division, Argonne National Laboratory, 9700 South Cass Avenue, Argonne, IL 60439 USA; 10 United States Department of Agriculture, Agricultural Research Service (USDA-ARS), Mandan, ND United States; 11 World Agroforestry, East and Central Asia, Heilongtan, Lanhei Road 132, Kunming, 650201 China; 12 Faculty of Forestry, University of Extremadura, Av. Virgen del Puerto, 2, 10600 Plasencia, Spain; 13 Department Of Soil Science, Federal University of Lavras, Main Campus, Lavras, Minas Gerais 37200-000 Brazil; 14 Centre for Ecology and Hydrology, Lancaster Environment Centre, Lancaster, LA1 4AP UK; 15 Natural Resource Ecology Laboratory, Colorado State University, Campus Delivery 1499, Fort Collins, Colorado 80523 USA; 16 CTFC Forest Science Centre of Catalonia, Solsona, Spain; 17 Mercator Research Institute on Global Commons and Climate Change (MCC), Torgauer Str. 12, 10829 Berlin, Germany; 18 Institute of Resource, Ecosystem and Environment of Agriculture, Nanjing Agricultural University, 1 Weigang, Nanjing, Jiangsu 210095 China; 19 Centre for Crop and Disease Management, Department of Environment and Agriculture, Curtin University, Perth, WA 6845 Australia

**Keywords:** Environmental impact, Ecosystem ecology, Agroecology, Ecosystem services

## Abstract

A global, unified dataset on Soil Organic Carbon (SOC) changes under perennial crops has not existed till now. We present a global, harmonised database on SOC change resulting from perennial crop cultivation. It contains information about 1605 paired-comparison empirical values (some of which are aggregated data) from 180 different peer-reviewed studies, 709 sites, on 58 different perennial crop types, from 32 countries in temperate, tropical and boreal areas; including species used for food, bioenergy and bio-products. The database also contains information on climate, soil characteristics, management and topography. This is the first such global compilation and will act as a baseline for SOC changes in perennial crops. It will be key to supporting global modelling of land use and carbon cycle feedbacks, and supporting agricultural policy development.

## Abstract (summary)

**Table:** 

Design Type(s)	data integration objective • data collection and processing objective • factorial design
Measurement Type(s)	amount of carbon atom in soil
Technology Type(s)	digital curation
Factor Type(s)	climate • Species • experimental condition • age
Sample Characteristic(s)	Brazil • cultivated environment • Canada • United States of America • New Zealand • Italy • Germany • Cameroon • India • Sri Lanka • Venezuela • Kingdom of Denmark • Republic of Ireland • French Republic • United Kingdom • Kingdom of Spain • Costa Rica • Ethiopia • Ghana • Indonesia • Mexico • Nigeria • Republic of South Africa • Portuguese Republic • China • Malaysia • Turkey • Israel • Sweden • italy • Georgia • Puerto Rico • Tanzania • Australia

Machine-accessible metadata file describing the reported data (ISA-Tab format)

## Background & Summary

Perennial crops are those crops that have a productive life cycle longer than a year, such as apple trees or sugarcane. Perennial crops comprise 30% of global croplands^1^. Despite their large spatial coverage, the effects of perennial crop plantations on soil properties are not fully understood and results from the literature to date are inconclusive. For example, studies have reported both soil organic carbon (SOC) higher^2,3^, lower^4,5^ or equal to^6^ after transition to perennial crops. This variation could be due to the diverse nature of published studies which have been carried out in different climates and soil types, with a wide range of crops and management practices. Furthermore, a variety of experimental designs, sampling strategies (frequency and soil depth) and analytical methods have also been used. As a result, studies are not directly comparable and conclusions on the effect of perennial crop cultivation on SOC stocks, and the underlying mechanisms, cannot be easily derived.

Accordingly, a major problem contributing to a lack of clear conclusions is that no harmonised data exist. The dataset we present fills that gap: we have compiled the largest dataset to date comprising studies which assess the effects on SOC and soil texture parameters of (a) temporal dynamics, e.g. presenting values across years (b) land use change to perennial crop, e.g. from annual crop or forest to perennial crop and (c) different management practices, e.g. soil amendments. We have included basic climate information: mean annual temperature and precipitation, and topography and bioclimatic region. We have also harmonised the data entry values. The process of harmonising data is to combine data from different and heterogeneous sources and formats into a single, consistent, integrated dataset. Information about SOC carbon stocks already exist and is available in repositories such as the harmonized world soil database^7,8^ from the Global Soil Information System (GLOSIS) or SoilGrids^9^. The dataset we present not only complements those SOC stock data but provides further information on SOC changes.

We have included different final end-use types of perennials: (i) food and beverage crops such as citrus or coffee, (ii) bioenergy crops such as *Miscanthus* or switchgrass, (iii) short rotation coppice trees such as poplar and willow, (iv) animal-feed such us *Brachiaria* or *Atriplex*, (v) agroforestry and (vi) other bio-products such as cotton or ramie.

**Fig. 1:**
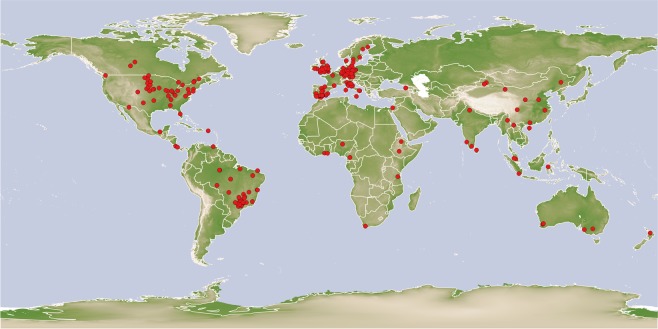
World map indicating the location of the plots included in the dataset in red dots.

The data are at figshare^10^. In total, the dataset contains information from 1605 ID entries in 180 studies, 709 field studies, 58 crops, from 32 countries in all continents except for Antarctica, covering all the bio-climatic areas suitable for agriculture (Fig. 1). The list of crops and countries included is in Online-only Table 1.Some areas are still under-represented but we hope that publication of this dataset will encourage those gaps to be filled. Every ID-entry is a paired-comparison point. The ID-entry contains either single SOC measurements, when possible, or aggregated data at plot level when it was not possible to obtain individual values. This has been clearly indicated in the dataset, as is the number of subsamples in the aggregated data specified, where these data were available. Data aggregated at larger scale, i.e. local or regional, was not considered. Each ID-entry contains information on SOC dynamics, which can be either temporal data or paired comparison data including the crop age and time since conversion to perennial, which can be used as a surrogate of temporal information (see methods for more details). For every ID-entry, the dataset contains: information from the original study on crop type, crop age, climate, basic physio-chemical soil characteristics and SOC, number of samples, the original study source, plus additional information about climate, soil properties and biogeographical regions from global databases (details in Online-only Table 2). We only included studies that have information about the perennial crop plantation establishment date and crop age. This was an essential condition since otherwise temporal carbon dynamics cannot be quantified. The aforementioned essential requirements required us to discard many papers reporting some SOC changes in perennial crops, most of which lacked information about crop age. A second common cause for discarding papers was the reporting of SOC change alone rather than as empirical pair-wise values. Aggregation at a larger scales was also frequent.

This harmonized dataset will be of value to support global modelling of land use change and carbon cycle feedbacks, to underpin development of climate-smart agricultural strategies, policy development for sustainable agriculture, to calculate potential carbon savings from global deployment of perennial bioenergy crops, and to support the 4 per mille initiative^11,12^, among others. Indeed, as the first of many potential analyses using these data, an empirical model of global patterns of SOC changes under perennial crops is being developed using these datasets.

## Methods

### Empirical data gathering

We compiled the dataset from a systematic search of the literature and by consulting researchers globally to identify and share available data. We furthermore searched in ISI Web of Knowledge, GoogleScholar and figshare using the keywords “perennial crop”, “soil organic carbon” and “soil organic carbon perennial”. We additionally followed references from papers identified in these searches and contacted some authors via email.

For every ID-entry, we added all the available data from the original study in the original units given in the study. This included information about location, climate, crop, soil characteristics, and number of soil samples, temporal/paired site data and original study source. Full details and a list of all entries are presented in Online-only Table 2. Besides, we classified the crops in three main groups: woody plants, palms and grasses (Online-only Table 1).

For ID-entry, two SOC and soil characteristics values are given. The first pair of the set is named “Current SOC and soil characteristic” and indicates the values of SOC at the most recent time point (for temporal data), the perennial land use (for paired site data including different land use) or new management versus traditional (for paired site data including different crop management). The paired SOC and soil characteristic values are under “Previous SOC and soil characteristic”.

### Extra fields added to the dataset: climate, topography, soil characteristics

We added additional values of climate variables, topography, soil characteristics and FAO ecoregion classification values from different global free-source datasets. In every case, we used the geographical coordinates given in the original sources to extract additional information. We did not use this information to gap-filling but provide it alongside in a complementary dataset. The data points can be matched and merged using the ID-entry.

Climate information for the plot was extracted from the WorldClim v2 dataset^13^, which uses average monthly climate data for 1970–2000 (http://www.worldclim.org/). In addition, we added a climatic water deficit index^14^, and calculated an indicator of water or climate stress P/(T + 10), where P is the annual precipitation and T the annual mean temperature. Details and units are presented in Online-only Table 3.

Information about soil texture and chemical properties was obtained from the SoilGrid Database^15^, which provides information at 250 m resolution at seven soil depths (https://soilgrids.org/#!/?layer=TAXNWRB_250m&vector=1). Details and units are given in Online-only Table 3.

To characterise the ecoregions, we used the FAO map of Eco-floristic regions (cdiac.ess-dive.lbl.gov/ftp/global_carbon/ecofloristic_zones.zip). Details and regions are given in Online-only Table 3.

Elevation information was extracted from the SRTM version 4.1, resolution approx. 250 m^16^, from the server http://srtm.csi.cgiar.org/SELECTION/inputCoord.asp. Using that elevation information, we created a digital elevation model, DEM using QGIS^17^ and derived the values of elevation, slope, aspect, hillshade and roughness at the plot coordinates. Details and units are given in Online-only Table 3. We also added the slope class from the FAO Agro-Ecological Zones (GAEZ v3.0), server (http://www.fao.org/geonetwork/srv/). The classes are: class 1, slope 0–2%; class 2: 2–5%; class 3: 5–8%; class 4: 8–16%; class 5: 16–30%; class 6: 30–45% and class 7: >45%.

### Unifying units

For soil properties, values of soil texture, clay, sand and silt were transformed to percentages if they were not in that unit in the original study. To do so, values of g/kg were divided by 10.

Values of soil bulk density (BD), texture and pH were not gap-filled but left as NA when not provided. This was to avoid masking which data were empirical and which data were not. Only 51% of the studies reported the values of BD, and 43% soil texture. But information on BD, texture and pH for each point can be found in the fields added to those obtained from the SoilGrids dataset, at the soil depth of the middle depth of the sample point. This information can be used to gap fill those fields if necessary.

Values of SOC in the original studies where either in concentration, g/kg, (*SOC*_*con*_) or in carbon stocks, Mg/ha (*SOC*_*stock*_). We initially considered transforming the values using the equation^18^:

$$SO{C}_{stock}=SO{C}_{con}\,\ast \,Bulk\,Density\,\ast \,depth\,\ast \,(1\,-\,rock\,fragments\,fraction).$$

However, that transformation would have added extra uncertainty and thus was not consistent with our principle of providing the SOC values as raw and close to empirical values as possible. Furthermore, when comparing the SOC values from transformed against untransformed data, we found a biased mismatch between the range of data using original units and the transformed data. We hypothesise that this may be due to the use of aggregated data or single use of BD for multiple samples^19,20^, or the use of different methodology^21^, or different sampling depth or soil core ring sizes, or lack of information about rock fragments. Considering this, we concluded that such a transformation was not appropriate, in line with previous reports^5^. Therefore, in this dataset, we provide SOC values using the units of the original study, which means about 30% are in g/kg (*SOC*_*con*_) and the other 70% in Mg/ha (*SOC*_*stock*_).

For climate variables, temperature and precipitation, the values reported in the original study were kept. Otherwise, we added the values obtained from the WorldClim dataset, which has a lag time of 30 years, averaging climate values from 1970 to 2000. The “temperature” and “precipitation” fields were the only ones that were gap-filled in the provided main database (in about 1% of the cases). Average values of temperature and precipitation were provided in most of the original studies, however, the period of time was specified hardly any case.

### Data aggregation quantification

We added a new field to indicate whether the original dataset contains information about a single SOC empirical measurement or aggregated data (in the “Data” section, Online-only Table 2). In addition, the entries “N plots” and “N samples” specify the number of empirical measurements per entry, when provided in the original studies. We added a new field called “N measured” which is the number of plots multiplied by the number of samples. Therefore, this value also indicates the level of data aggregation. When only the number of plots or samples per plot were indicated in the original study, this was the value given in this field. When neither of these values were reported in the original study, the number of measurements was set to 1. In the dataset, 108 data-points contained a single measurement, 965 ID-entries were aggregated data, and for the remaining 532 samples no details of aggregation were provided. From the 965 entries with aggregated data, only 319 (33%) reported the standard deviation or variation in the original study.

## Data Records

The dataset, in spreadsheet format, can be found in the figshare repository^10^ with the title “A global dataset of harmonised empirical values of soil organic carbon changes under perennial crops v1.0”

The creators are: Alicia Ledo, Jonathan Hillier, Pete Smith, Eduardo Aguilera, Sergey Blagodatskiy, Francis Q. Brearley, Ashim Datta, Eugenio Diaz-Pines, Axel Don, Marta Dondini, Jennifer Dunn, Diana Marisa Feliciano, Mark A. Liebig, Rong Lang, Mireia Llorente, Yuri Lopes Zinn, Niall McNamara, Stephen Ogle, Zhangcai Qin, Pere Robira, Rebecca Rowe, José Luis Vicente Vicente, Jeanette Whitaker, Qian Yue, Ayalsew Zerihun.

**Fig. 2:**
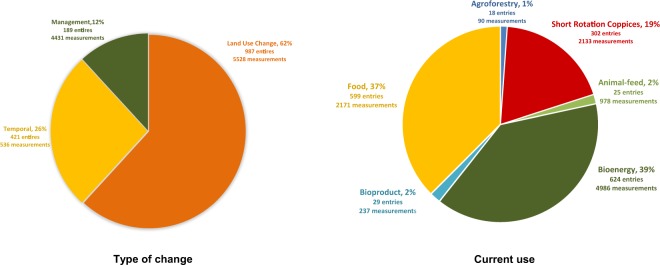
Number of entries and measurements in the dataset according to type of change and current use.

The list of crops and countries is presented in Online-only Table 1. A summary Table summarising the different types of data included is in Table 1, and the percentage of data points in each category can be seen in Fig. 2.

**Table 1:** Summary of the data included in the dataset according to: (i) type of dynamic data (ii) aims of the original study (iii) types of data aggregation (iv) end-use of perennial crop.

Type of data	Explanation
**Types of dynamic data**	
Temporal data	Same site, two or more different timepoints
Paired site data to assess land use change	Same timepoint, adjacent sites with non-perennial and perennial crop land use
Paired site data to assess management	Same timepoint, adjacent sites with different crop management
**Aims of the original study**	
Temporal changes in Soil Organic Carbon under perennial crops during the crop life cycle	
Changes in Soil Organic Carbon after land use change, from different systems to perennial crops	
Changes in Soil Organic Carbon after a change in management, e.g. from intensive to organic farming	
**Types of data aggregation**	
Data from single points	Each entries is a single measurement at a particular depth
Data already aggregated in the original study	Multiple samples at a particular plot
**End-use of the current perennial crop**	
Animal-feed	Crops used to feed
Bioenergy grasses	Such us *Miscanthus*
Bio-products	Other bio products such as cotton
Food crops	Including both food and drink products such as apples and coffee respectively
Short rotation coppice	Used for wood or bioenergy
Agroforestry	Agroforestry

Metadata information in ISA format (www.isa-tools.org) is provided in the associated Scientific Data Descriptor. The dataset we present is compliant with the FAIR principles for scientific data^22^: findable, accessible, intraoperative and reusable.

## Technical Validation

### Variable distribution and unusual point values

We evaluated the distribution of all the quantitative variables. We examined the outliers and/or extreme points for potential errors. The distribution of all variables can be found in Supplementary Material 1 (Scatterplots, box-and-whiskers and histograms).

### Quantifying errors and standard deviations in SOC

**Fig. 3:**
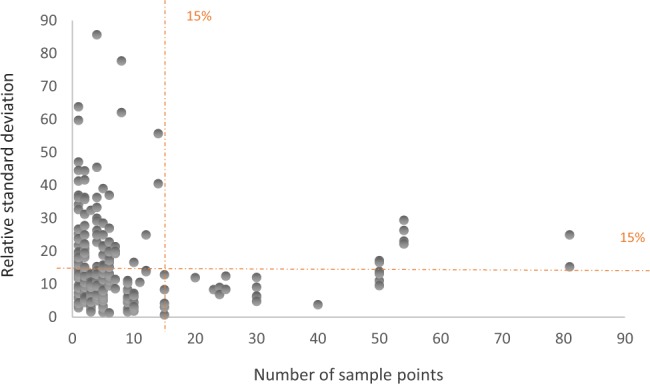
Relative standard deviations of percentage of soil organic carbon against the number of samples per plot taken. Each grey dot corresponds to a plot. The orange dashed lines mark the 15%.

We analysed the effect of the number of samples per plot in terms of uncertainty. To this end, we calculated the relative standard deviation (sd) of the SOC mean values for ID-entries with more than one measurement and reporting the sd of SOC values. Overall, the value of relative standard deviation of SOC mean values, sd/SOC, which is indeed the coefficient of variation (cv), decreased as the number of samples increased (Fig. 3). In other words, a greater sampling effort seems to decrease the cv. According to these data, the relative standard deviation is generally under 10–15% after gathering 20 sample points. Nonetheless, this depends on the soil characteristics and the sampling spatial distribution. All in all, having 20–30 replicates per plot seems to be an adequate sample size in SOC studies to constrain the coefficient of variation below 15% (Fig. 3).

### On the SOC errors

Assessing SOC stocks is a complex process which involves several steps and measurements. It should thus be borne in mind that many types of error may have crept in the original data acquisition. Besides, while some errors may be random, others may be systematic thus introducing bias, the latter is of a greater concern and efforts should be made to minimise it. Not to mention that assessing SOC stocks is a complex matter itself. The dataset may contain different types of errors from different sources. For example measurement errors in the field, lab analytical and measurement errors and post-process data errors and transformations are all possible. A further description and comments on SOC errors is beyond the scope of this data paper and can be found elsewhere, e.g.^18,19,23–26^ among others.

SOC measurement and stock estimation also usually involve measuring or estimating other soil parameters, namely BD, and SOC concentration and determination accuracy are often affected by other properties such as texture and iron oxide contents. Mathematical relationships to link those parameters may introduce further bias^18^. However, measurement and analytical errors can lead to both under- and over-estimation. If this is the case and the errors are randomly distributed, they somewhat compensate for each other in large datasets. Non-random, systematic errors, may also mask trends in the data. However, it is not possible to know to which extent these kind of errors are present in the SOC perennials dataset given the available source information. In any case, we recommend quantifying sources of uncertainty when possible, and at different temporal and spatial scales^23^ and due to data aggregation^19^.

Different sampling depth is another potential source of error and uncertainty when comparing data from different studies^27^. In this dataset we have brought together SOC at different depths (the sample depth is clearly indicated in the dataset), but we suggest that values of SOC measured at different depths should not be mixed without using a suitable pedo-transfer function (PTF). Presenting new PTF for perennials is beyond the scope of this data paper yet is currently being explored for future publication.

### A call for unification in reporting soil properties data

An increasing number of studies^18,19,21^ are reporting problems in comparing SOC data, and some of the given reasons have been mentioned in the previous subsection. Values of bulk density and soil texture are measured in most studies but only at times reported in the resulting manuscript. This first source of uncertainty, and potential errors and bias, can be remedied by including this information in publications. Thus, we formally appeal to researchers to measure and report BD and soil texture in future SOC studies. BD is technically simpler to measure than texture, which is a time consuming analytics and could be constrained by budget and or time. However, easy methods to estimate soil texture directly in the field have been proposed and evaluated as accurate enough for most purposes^28^.

A second essential piece of information not always detailed in published papers is the number of samples and replicates. We encourage researchers to provide all of the relevant data and measurements done, and for reviewers to request that authors provide this information, for the sake of transparency, reproducibility and quantification of uncertainty.

A particular drawback in current SOC studies is the lack of standardised carbon values to be reported. Until now, some studies use SOC concentration values (g/kg), while others use carbon stocks (Mg/ha) or volumetric values (g/m^3^). This is not a matter of different units, since those carbon pools are different things. Nonetheless, they are interchangeable if and only if the required soil variables are accurately measured and provided (SOC content, BD, soil texture, rock fragment, maximum depth), as we suggested and discussed in the previous sections. This lack of consensus and unification is not only limiting values of some relevant studies^5^, but is hampering our understanding of soil carbon dynamics. It is timely that we gather and unify knowledge to better understand of the carbon cycle in general, and SOC dynamics in particular. This understanding is key to realistic estimation of the potential of SOC storage in perennial crops as a climate change mitigation option, and a potential tool to deliver the goals of the 4 per 1000 initiative. This knowledge is also important to predict potential changes on SOC dynamics under climate change conditions and to underpin development of climate-smart agricultural strategies.

The ISRIC WDC *GlobalSoilMap* from the World Soil Information Service (WoSIS) has recently provided some standards and procedures to acquire, harmonise and provide soil data^7,29^. In further SOC analysis and work, it may be adequate to align with those protocols.

The minimum information we recommend that should be provided in SOC studies is: (i) number of plots and number of replicates per plot (ii) depth of measurement and number of layers considered, (iii) BD and equivalent soil mass, (iv) soil texture, (v) rock fragment fractions (vi) SOC in concentration and stocks, (vii) method used to measure SOC and BD, (viii) climate, or at least mean annual temperature, precipitation and elevation, (ix) soil management (if any), and (x) land use (historic land use is also highly desirable). The WoSIS repository has a shorter list of requirements. Nevertheless, to align with its standards, cation exchange capacity, electrical conductivity and water holding capacity are also required^29^.

## Supplementary Information

### ISA-Tab metadata file

Download metadata file

### Supplementary information

Supplementary Material 1

## Online-only Tables

**Online-only Table 1:** List of crops and locations included in the dataset.

Crop latin name	Common name	Type	Country/es
*Acacia* sp.	Acacia	Woody plant	Tanzania
*Malpighia emarginata* DC.	Acerola	Woody plant	Brazil
*Alnus* sp.	Alder	Woody plant	Germany
*Prunus dulcis* (Mill) D.A. Webb	Almond	Woody plant	Spain, Italy
*Bixa Orellana* L.	Annato	Woody plant	Brazil
*Annona Guanabanus* Mill.	Annona	Woody plant	Brazil
*Prunus armeniaca* L.	Apricot	Woody plant	Italy
*Araucaria angustifolia Bertol*. (*Kunze*)	Araucaria	Woody plant	Brazil
*Areca catechu* L.	Arecanut	Palm	India
*Atriplex* sp.	Atriplex	Woody plant	Australia
*Paspalum notatum* Flüggé	Bahiagrass	Grass	USA
*Musa* sp.	Banana	Palm	Sri Lanka, Costa Rica, China, Brazil
*Robinia pseudoacacia* L.	Black locust	Woody plant	Italy, Germany
*Vaccinium* sp.	Blueberry	Woody plant	USA
*Brachiaria eruciformis* (Sm.) Griseb	Brachiaria	Grass	Venezuela
*Bertholletia excelsa* Humb. & Bonpl.	Brazil nut	Woody plant	Brazil
*Anacardium occidentale* L.	Cashew	Woody plant	Brazil
*Casuarina* sp.	Casuarina	Woody plant	Puerto Rico
*Toona ciliata M*. *Roem*	Australian red cedar	Woody plant	Brazil
*Citrus* sp.	Citrus	Woody plant	Italy
*Theobroma cacao* L.	Cocoa	Woody plant	Cameroon, Brazil, Ghana, Indonesia, Costa Rica
*Cocos nucifera* L.	Coconut	Palm	Brazil
*Coffea arabica* L.	Coffee	Woody plant	Brazil
*Gossypium arboretum* L.	Cotton	Woody plant	USA, China
*Cucumis sativus* L.	Cucumber	Grass	China
*Theobroma grandiflorum* (Willd. ex Spreng.) K.Schum.	Cupuacu	Woody plant	Brazil
*Eucalyptus* sp.	Eucalyptus	Woody plant	Brazil, Puerto Rico, Australia, India
*Arundo donax* L.	Giant reed	Grass	Italy
*Psidium guajava* L.	Guava	Woody plant	Brazil, India
*Syzygium cumini* (L.) Skeels	Cloves	Woody plant	India
*Jatropha sp*.	Jatropha	Woody plant	India, Brazil
*Actinidia chinensis* Planch	Kiwi	Woody plant	New Zealand, Italy
*Litchi chinensis* Sonn.	Lychee	Woody plant	India
*Zea mays* L.	Maize	Grass	Germany
*Mangifera indica* L.	Mango	Woody plant	Brazil, India
*Manilkara* sp.	Manilkara	Woody plant	Brazil
*Cucurbita* sp.	Melon	Grass	China
*Miscanthus* sp.	*Miscanthus*	Grass	Italy, Germany, Denmark, Ireland, France, USA, UK
*Morus* sp.	Mulberry	Woody plant	China
*Olea europaea* L.	Olive	Woody plant	Spain, Italy, Portugal
*Citrus* sp.	Orange	Woody plant	Spain
*Elaeis guineensis* Jacq.	Oil palm	Palm	Brazil, Malaysia
*Prunus persica* (L.) Batsch	Peach	Woody plant	Brazil
*Arachis hypogaea* (L.)	Peanut	Woody plant	USA
*Pinus* sp.	Pine	Woody plant	Brazil
*Populus* sp.	Poplar	Woody plant	Canada, Italy, Germany, USA
*Prosopis* sp.	*Prosopis*	Woody plant	India
*Boehmeria nivea* (L.) Gaudich.	Ramie	Grass	Italy
*Hevea brasiliensis* Müll. Arg.	Rubber	Woody plant	Ghana, China
*Lolium* sp.	Ryegrass	Grass	Germany
*Picea* sp.	Spruce	Woody plant	Canada
—	Stone fruits	Woody plant	Israel
*Saccharum officinarum* L.	Sugarcane	Grass	Brazil, Mexico, China
*Panicum virgatum* L.	Switchgrass	Grass	Italy, USA, Germany, Canada
*Camellia sinensis* (L.) Kuntze	Tea	Woody plant	China, Georgia
*Vitis vinifera* L.	Grapes	Woody plant	Spain, France, South Africa, Italy, USA, Turkey
*Salix* sp.	Willow	Woody plant	Italy, Germany, Canada, England, UK, Sweden

**Online-only Table 2:** List of the column entries in the dataset, including section subsection and a brief description.

Section	Subsection	Description*
ID	ID	Entry identifier, ID-entry
	ID study	Study identifier
	plot ID	Plot identifier
SITE	Country	Country
	Region	Region
	Temperature	Temperature, Celsius
	Precipitation	Annual precipitation, mm
	Climate	Climate type
	Latitude	Latitude, degrees
	Longitude	Longitude, degrees
	Bedrock	Bedrock type
	Soil type	Soil classification according to FAO
	USDA	Soil classification according to USDA
TYPE CHANGE	Type change	TEMP (temporal data within crop life), LUC (land use change to perennial) or MANAG (change in management), LUC REVERSE (land use change from perennial to annual crop)
CROP AND LAND USE	CROP current	Crop
	CROP type	Main crop classification: grass (for perennial grasses), woody (for woody plants), palm (for palm trees)
	crop AGE	Crop age
	Current Land Use	agro for (agroforestry system), animal-feed, annual crop, bioenergy (bioenergy grass), bioproduct, food, SCR (short rotation coppice).
	LUC this study	Whether this study includes any land use change or not: YES or NO
	Previous land use	annual crop, fallow, forest, grassland, natural forest, pasture, pasture + annual crop, peatland, perennial food, secondary forest, SRC
	Years since LUC	Years since land use change
	Temporal only this study	Whether this study includes only temporal data: YES or NO
	Time temporal	Time lag for the temporal change
SOIL MANAGEMENT INFO	CHANGE management	Whether this study includes different management options of a crop or not: YES or NO
	Year since management change	Year since change
	Soil treatments	YES or NO
	Tillage YES NO	YES or NO
	Tillage type	Deep, medium, mowing, normal, reduced, shallow, subsoil, year 0,YES, NO, NA
	Fertilizer YES NO	Whether any kind of fertiliser is applied or not
	Fert type	Fertilizer type (see the main dataset for details)
	Rate fertilizer	fertilizer rate, kg ha^−1^
	Pesticides	Whether any kind of pesticide is applied or not
	org amend YES NO	YES or NO
	org amend type	Organic amend type (see the main dataset for details)
	prev man	Previous management: organic, normal, intensive, NA
DATA	SINGLE DATA-MULTIPLE SAMPLING	MULTI (data have been aggregated) SINGLE (no aggregated data)
	DIF DEPTH current point	YES or NO, indicating whether the study contains information of SOC at different depths on the same point or not.
	CONTI_DEPTH	YES or NO. If yes, the different depths are continuous, i.e. 0–20, 20–100. If not, all the values are from 0 to the depth, i.e. 0–20, 0–100.
	NUM_DEPTH	Number of different depth layers provided
CURRENT SOC AND SOIL CHARACTERISTICS	year measure	Year the soil samples were taken
	N plots current	Number of plots
	N sample per plot current	Number of samples per plot
	N measured current	Number of plots multiplied by the number of samples
	Soil from cm current	Soil depth from, in cm
	Soil to cm current	Soil depth to, in cm
	Depth midpoint current	Soil depth midpoint, in cm
	Bulk density Mg m^3^ current	Bulk density, in Mg m^−3^
	% clay current	Percentage of clay
	% silt current	Percentage of silt
	% sand current	Percentage of sand
	pH current	pH
	SOC g kg current	Current Soil organic Carbon concentration, in g kg^−1^
	sd SOC g kg current	Standard deviation of the SOC mean values, in g kg^−1^
	SOC Mg ha current	Current Soil organic Carbon stochs, in Mg ha^−1^
	sd SOC Mg ha current	Standard deviation of the SOC mean values, in Mg ha^−1^
PREVIOUS SOC AND SOIL CHARACTERISTICS	N plots previous	Number of plots
	N sample per plot previous	Number of samples per plot
	N measured previous	Number of plots multiplied by the number of samples
	Soil from cm previous	Soil depth from, in cm
	Soil to cm previous	Soil depth to, in cm
	Depth midpoint previous	Soil depth midpoint, in cm
	Bulk density previous	Bulk density, in Mg m^−3^
	% clay previous	Percentage of clay
	% silt previous	Percentage of silt
	% sand previous	Percentage of sand
	pH previous	pH
	SOC g kg previous	Previous Soil organic Carbon concentration, in g kg^−1^
	sd SOC previous	Standard deviation of the SOC mean values, in g kg^−1^
	SOC Mg ha previous	Previous Soil organic Carbon stochs, in Mg ha^−1^
	sd SOC previous	Standard deviation of the SOC mean values, in Mg ha^−1^
SOURCE	yearPpub	year paper published
	original source	original paper full reference

*NA means that value was not available or unknown.

**Online-only Table 3:** List and details of the extra climate, soil and geographical variables added to the original dataset; indicating the database source and including the units and spatial and temporal resolution.

Dataset	Variable	Units	Spatial Resolution	Temporal resolution	Name in database
SRTM	Elevation	Meters above sea level	30 m		elevation
QGIS (STMR)	Slope	Angle	30 m		slope
	Aspect		30 m		aspect
	Hillshade		30 m		Hillshade
	Roughness		30 m		roughness
World Clim v2	Solar radiation per month	KJ/m^2^	30 seconds (~1 km^2^)	Averaged monthly 1970–2000	‘SolarRad_Jan’ to ‘SolarRad_Dec’
	Bio 1: Annual Mean Temperature	Celsius	30 seconds (~1 km^2^)	Annual Mean Temperature	Bio1
	Bio 2: Mean Diurnal Range (Mean of monthly (max temp - min temp))	—	30 seconds (~1 km^2^)	Annual	Bio2
	Bio 3: Isothermality (BIO2/BIO7) (*100)	—	30 seconds (~1 km^2^)	Annual	Bio3
	Bio 4: Temperature Seasonality (standard deviation *100)	Celsius	30 seconds (~1 km^2^)	Annual	Bio4
	Bio 5: Max Temperature of Warmest Month	Celsius	30 seconds (~1 km^2^)	Annual	Bio5
	Bio 6: Min Temperature of Coldest Month	Celsius	30 seconds (~1 km^2^)	Annual	Bio6
	Bio 7: Temperature Annual Range (BIO5-BIO6)	—	30 seconds (~1 km^2^)	Annual	Bio7
	Bio 8: Mean Temperature of Wettest Quarter	Celsius	30 seconds (~1 km^2^)	Annual	Bio8
	Bio 9: Mean Temperature of Driest Quarter	Celsius	30 seconds (~1 km^2^)	Annual	Bio9
	Bio 10: Mean Temperature of Warmest Quarter	Celsius	30 seconds (~1 km^2^)	Annual	Bio10
	Bio 11: Mean Temperature of Coldest Quarter	Celsius	30 seconds (~1 km^2^)	Annual	Bio11
	Bio 12: Annual Precipitation	mm	30 seconds (~1 km^2^)	Annual	Bio12
	Bio 13: Precipitation of Wettest Month	mm	30 seconds (~1 km^2^)	Annual	Bio13
	Bio 14: Precipitation of Driest Month	mm	30 seconds (~1 km^2^)	Annual	Bio14
	Bio 15: Precipitation Seasonality (Coefficient of Variation)	mm	30 seconds (~1 km^2^)	Annual	Bio15
	Bio 16: Precipitation of Wettest Quarter	mm	30 seconds (~1 km^2^)	Annual	Bio16
	Bio 17: Precipitation of Driest Quarter	mm	30 seconds (~1 km^2^)	Annual	Bio17
	Bio 18: Precipitation of Warmest Quarter	mm	30 seconds (~1 km^2^)	Annual	Bio18
	Bio 19: Precipitation of Coldest Quarter	mm	30 seconds (~1 km^2^)	Annual	Bio19
Chave *et al*. (2015)	Climate water deficit (CWD)	—		Annual	CWD
P/(T + 10)	Bio12/(Bio 1 + 10)	mm/Celisus	30 seconds (~1 km^2^)	Annual	PT10
Soil Grids	Absolute depth to bedrock	cm	250 m	—	BedDepth
	Bulk density at 7 different depths*	Kg/m^3^ of the fine earth fraction (<2 mm)	250 m	—	‘BD_1’ to ‘BD_7’
	pH in H_2_O at 7 different depths*	pH in H_2_O	250 m	—	‘pH_1’ to ‘pH_7’
	Clay at 7 different depths*	%	250 m	—	‘clay_1’ to ‘clay_7’
Soil Grids	Silt at 7 different depths*	%	250 m	—	‘Silt_1’ to ‘Silt_7’
	Sand at 7 different depths*	%	250 m	—	‘Sand_1’ to ‘Sand_7’
	Cation-exchange capacity at 7 different depths*	Cmol+/kg of the fine earth fraction (<2 mm)	250 m	—	‘CAT_1’ to ‘CAT_7’
	Coarse fragments volumetric fraction at 7 different depths*	%	250 m	—	‘CVOL_1’ to ‘CVOL_7’
	Available soil water capacity (volumetric fraction) until wilting point at 7 different depths*	%	250 m	—	‘WatCap_1’ to ‘WatCap_7’
	Available soil water capacity (volumetric fraction) for h1 at 7 different depths*	%	250 m	—	‘WCat1h_1’ to ‘WCat1h_7’
	Saturated water content (volumetric fraction) for tS at 7 different depths*	%	250 m	—	‘SatWat_1’ to ‘SatWat_7’
	Soil organic carbon density in kg per cubic-m at 7 different depths*	kg per cubic-m	250 m	—	‘GRIDSOC_kgm3_1’ to ‘GRIDSOC_ kgm3_7’
	Soil organic carbon stock in tons per ha at 6 different depths* plus three extra depths (30 to 250 m; 100 to 250 m and 200 to 250 m)	tons per ha	250 m	—	‘GRIDSOC_tonha_1’ to ‘GRIDSOC_tonha _6’ and ‘GRIDSOC_tonha _30_250’, ‘GRIDSOC_tonha _100_250’, ‘GRIDSOC_tonha _200_250’
	Soil organic carbon content (fine earth fraction) in g per kg at 7 different depths*	%	250 m	—	‘GRIDSOC_gKg_1’ to ‘GRIDSO_gKg _7’
FAO	Slope class	class	30 m		Slope Class*
FAO	Eco regions	—	—		Ecoregion*

*Slope Class: class 1, slope 0–2%; class 2: 2–5%; class 3: 5–8%; class 4: 8–16%; class 5: 16–30%; class 6: 30–45% and class 7: >45%.

*Ecoregions: Tropical moist deciduous forest, Boreal coniferous forest, Temperate steppe, Subtropical humid forest, Temperate oceanic forest, Tropical rainforest, Tropical shrubland, Tropical dry forest, Subtropical dry forest, Temperate desert, Subtropical steppe, Temperate continental forest, Tropical mountain system, Tropical desert, Temperate mountain system, Subtropical desert, Water, Tropical shubrland.

*Depths (following to the Soil Grids classification): class 1, 0 cm; class 2, 5 cm; class 3, 15 cm; class 4, 30 cm; class 5, 60 cm; class 6, 100 cm and class 7 200 cm.

## References

[1] Ledo A, Heathcote R, Hastings A, Smith P, Hillier J (2018). Perennial-GHG: A new generic allometric model to estimate biomass accumulation and greenhouse gas emissions in perennial food and bioenergy crops. Environ. Modell. Softw..

[2] Lal R (2004). Soil carbon sequestration impacts on global climate change and food security. Science.

[3] Post WM, Kwon KC (2000). Soil carbon sequestration and land-use change: processes and potential. Global Change Biol..

[4] Crowther TW (2016). Quantifying global soil carbon losses in response to warming. Nature.

[5] Don A, Schumacher J, Freibauer A (2011). Impact of tropical land-use change on soil organic carbon stocks–a meta-analysis. Global Change Biol.

[6] Qin Z, Dunn JB, Kwon H, Mueller S, Wander MM (2016). Soil carbon sequestration and land use change associated with biofuel production: empirical evidence. GCB Bioenergy.

[7] Batjes, N. H. Overview of procedures and standards in use at ISRIC WDC-Soils. Report2017/01, ISRIC–World Soil Information, Wageningen, 10.17027/isric-wdcsoils.20170001 (2017).

[8] Nachtergaele, F. *et al*. The harmonized world soil database. In *Proceedings of the* 19th *World Congress of Soil Science*, *Soil Solutions for a Changing World*, *Brisbane*, *Australia*, 1–6 August 2010 (2010).

[9] Hengl T (2014). Soil Grids 1 km—global soil information based on automated mapping. Plos One.

[10] Ledo, A. *et al*. A global dataset of harmonised empirical values of soil organic carbon changes. *Figshare*, 10.6084/m9.figshare.7637210 (2019).

[11] The 4 per 1000 Initiative, https://www.4p1000.org/ (2016).

[12] Francaviglia, R., Di Bene, C., Farina, R., Salvati, L. & Vicente-Vicente, J. L. Assessing “4 per 1000” soil organic carbon storage rates under Mediterranean climate: a comprehensive data analysis. *Mitigation and Adaptation Strategies for Global Change*, 10.1007/s11027-018-9832-x (2019).

[13] Fick SE, Hijmans RJ (2017). WorldClim 2: new 1-km spatial resolution climate surfaces for global land areas. International Journal of Climatology.

[14] Chave J (2015). Improved allometric models to estimate the aboveground biomass of tropical trees. Global Change Biol..

[15] Hengl T (2017). SoilGrids250m: Global gridded soil information based on machine learning. Plos One.

[16] Jarvis, A., Reuter, H. I., Nelson, A. & Guevara, E. *Hole-filled SRTM for the globe version 4*, *from the CGIAR-CSI SRTM 90 m database*, http://srtm.csi.cgiar.org (2008).

[17] QGIS Development Team. *QGIS Geographic Information System*, http://qgis.osgeo.org (2018).

[18] Poeplau C, Vos C, Don A (2017). Soil organic carbon stocks are systematically overestimated by misuse of the parameters bulk density and rock fragment content. Soil.

[19] Grosz B (2017). The implication of input data aggregation on up-scaling soil organic carbon changes. Environ. Modell. Softw..

[20] Wendt J, Hauser S (2013). An equivalent soil mass procedure for monitoring soil organic carbon in multiple soil layers. Eur. J. Soil Sci..

[21] Lee J, Hopmans JW, Rolston DE, Baer SG, Six J (2009). Determining soil carbon stock changes: simple bulk density corrections fail. Agr. Ecosyst. Environ..

[22] Wilkinson, M. D. *et al*. The FAIR Guiding Principles for scientific data management and stewardship. *Scientific Data* **3** (2016). 10.1038/sdata.2016.18 PMC4792175 26978244

[23] Goidts E, Van Wesemael B, Crucifix M (2009). Magnitude and sources of uncertainties in soil organic carbon (SOC) stock assessments at various scales. Eur. J. Soil Sci..

[24] Jones, C. & Falloon, P. Sources of uncertainty in global modelling of future soil organic carbon storage. In *Uncertainties in Environmental Modelling and Consequences for Policy Making* (2009).

[25] Kutsch, W. L., Bahn, M. & Heinemeyer, A. *Soil carbon dynamics: an integrated methodology*. (Cambridge University Press, 2009).

[26] Stockmann U (2013). The knowns, known unknowns and unknowns of sequestration of soil organic carbon. Agr. Ecosyst. Environ..

[27] VandenBygaart A (2011). Impact of sampling depth on differences in soil carbon stocks in long-term agroecosystem experiments. Soil Sci. Soc. Am. J..

[28] Vos C, Don A, Prietz R, Heidkamp A, Freibauer A (2016). Field-based soil-texture estimates could replace laboratory analysis. Geoderma.

[29] Batjes NH (2017). WoSIS: providing standardised soil profile data for the world. Earth Syst. Sci. Data.

