# Supplementary material for: A global, empirical, harmonised dataset of soil organic carbon changes under perennial crops
Source: Sci Data. 2019 May 13;6:57. doi: 10.1038/s41597-019-0062-1 (PMC6514006; doi:10.1038/s41597-019-0062-1)
Supplement: Supplementary file 2 — Supplementary Material 1 [file 41597_2019_62_MOESM2_ESM.docx]

### Supplementary material

Supplementary Material 1: For each variable included in the dataset: (a) Scatter plot (b) boxplot showing the average, interquartile range and range of inorganic soils, and (c) histogram showing the values frequency.

NOTE: Organic soils were not included in the SOC boxplot, to have a better display
